# Evaluation of an Adaptive Dynamic Compensation System in Cochlear Implant Listeners

**DOI:** 10.1177/2331216520970349

**Published:** 2020-12-28

**Authors:** Florian Langner, Andreas Büchner, Waldo Nogueira

**Affiliations:** 1 Department of Otolaryngology, Medical University Hannover and Cluster of Excellence Hearing4all, Hanover, Germany

**Keywords:** cochlear implants, compression, dynamic range, speech intelligibility

## Abstract

Cochlear implant (CI) sound processing typically uses a front-end automatic gain control (AGC), reducing the acoustic dynamic range (DR) to control the output level and protect the signal processing against large amplitude changes. It can also introduce distortions into the signal and does not allow a direct mapping between acoustic input and electric output. For speech in noise, a reduction in DR can result in lower speech intelligibility due to compressed modulations of speech. This study proposes to implement a CI signal processing scheme consisting of a full acoustic DR with adaptive properties to improve the signal-to-noise ratio and overall speech intelligibility. Measurements based on the Short-Time Objective Intelligibility measure and an electrodogram analysis, as well as behavioral tests in up to 10 CI users, were used to compare performance with a single-channel, dual-loop, front-end AGC and with an adaptive back-end multiband dynamic compensation system (Voice Guard [VG]). Speech intelligibility in quiet and at a +10 dB signal-to-noise ratio was assessed with the Hochmair–Schulz–Moser sentence test. A logatome discrimination task with different consonants was performed in quiet. Speech intelligibility was significantly higher in quiet for VG than for AGC, but intelligibility was similar in noise. Participants obtained significantly better scores with VG than AGC in the logatome discrimination task. The objective measurements predicted significantly better performance estimates for VG. Overall, a dynamic compensation system can outperform a single-stage compression (AGC + linear compression) for speech perception in quiet.

Automatic gain control (AGC) is an essential component in many areas of audio digital signal processing, such as music mixing, radio, television transmission, and hearing aids, which adapts the large dynamic range (DR) of the original sound to the smaller DRs of the sound reproduction system. This process normally results in the softest parts of a signal becoming louder and/or the loudest parts become softer, implying a reduction/compression of the DR for a given sound. In hearing devices, AGC can help to address the more limited DR of people with hearing impairment, including people with cochlear implants (CIs). CIs can only use an electric DR of about 10 to 20 dB (Skinner et al., 1997; Zeng et al., 2002; Zeng & Galvin, 1999). A reduction of the acoustic DR of an incoming signal is a logical step for this small perception range. However, if temporal features, such as the minimum and maximum amplitude, can be extracted and used to adaptively change the signal processing, intelligibility and general sound perception might benefit.

The AGC can — if designed poorly — also introduce distortions of the temporal envelope of a signal, creating unnatural sounding transitions (Stone et al., 1999). It is however important to note that the instantaneous compression that is applied at the electrode mapping of CIs distorts the temporal envelope even more strongly than the AGC (Zeng & Galvin, 1999). The electrode mapping is the last step of the signal processing, converting the acoustically analyzed envelope magnitudes of the signal to the individual electrode currents to stimulate the auditory nerve. Both the AGC and the electrode mapping can affect speech intelligibility and sound perception in general. Back-end processing schemes (performed after the frequency analysis of the different bands) such as Adaptive Dynamic Range Optimization (ADRO; James et al., 2002) and the envelope profile limiter (Khing et al., 2013b) attempt to circumvent these problems by adjusting the gain in individual frequency channels of the signal, with the envelope profile limiter being a fast multichannel compressor with infinite compression above threshold. For both schemes, the DR of each frequency channel is defined by three target levels (rules) that represent comfort, background, and audibility to keep the output level within the audible-to-comfortable range (James et al., 2002). If the sound environment is composed of sounds mainly below 40 dB sound pressure level (SPL), more gain is required to stimulate within the threshold and comfortable range. Conversely, for high-level sounds, a reduction in gain would be required to keep the loudness levels within the threshold and comfortable levels. The gain rules use percentile estimates of the long-term output level for each band. Peak levels are represented by the 98^th^ percentile level, that is, the level that is exceeded only 2% of the time. For the average level, the 70^th^ percentile rule was used, and for the background noise level, the 40^th^ percentile level is used. These levels are computed approximately every 2 ms using a traditional percentile level estimator (James et al., 2002). The time constants for these estimates are relatively slow at around 20 dB per second, which is the time constant used by the integrator defined in the level estimator (Nysen, 1980). Some studies have shown significant improvements of speech intelligibility in quiet and noise as well as user preferences for ADRO (Blamey, 2005; Blamey et al., 2005; Dawson et al., 2004). These findings suggest that a regular, front-end, single-channel AGC processing is not an ideal solution for the everyday use of a hearing aid or CI, while back-end processing solutions such as ADRO might improve performance. However, a more recent study indicated that the improvements made with ADRO and the envelope profile limiter cannot be reproduced when tested in more realistic acoustic environments. Today, all the commercial CI systems of Cochlear Ltd. (Sydney, Australia) employ the ADRO scheme.

Speech has a range of levels of 30 to 40 dB around the mean speech level (ANSI, 2018; Studebaker et al., 1999). Moreover, it has been shown that this range of levels around the mean speech level is sufficient for speech perception in quiet for normal-hearing and hearing-impaired listeners, and an even narrower DR is sufficient for speech perception in noise (Stone et al., 2010). Hence, it is highly unlikely that the preservation of a larger range around the mean level is necessary in real-world scenarios for normal-hearing and hearing-impaired listeners. However, the overall range of all speech levels (input dynamic range [IDR] minimum and maximum level of the input signal) can be as much as 70 dB in the broadband signal. For CI users, Spahr et al. (2007) suggested that the intelligibility of speech at conversational levels in quiet and noise as well as speech at soft levels in quiet can be improved if the IDR of each frequency channel is larger than 30 dB. Zeng et al. (2002) also investigated the effect of IDR of each frequency channel on phoneme recognition in CI users and found an optimal IDR of around 50–60 dB. In addition, low-frequency sounds may dominate the IDR of the broadband signal, and therefore, in high frequencies, a higher DR in CI signal processing may be required than suggested in the ANSI standard. A recent study also found difficulties with the Speech Intelligibility Index, characterized in the ANSI standard, to predict speech intelligibility of CI users due to their highly variable outcomes (Lee et al., 2019).

The results using the ADRO strategy and the studies by Spahr et al. (2007) and Zeng et al. (2002) suggest that a larger IDR could be more desirable in CI signal processing than a smaller IDR with AGC processing. A larger IDR would capture the parts of the signal with the highest content of information and use these for further processing. ADRO uses a fast Fourier transform (FFT) to capture small spectral differences in speech represented, for example, in logatomes. These nonsense words used for syllable intelligibility testing center around a vowel (e.g., “bAb,” “gAg”) or consonant (e.g., “aTa,” “aFa”) and are significantly easier to discriminate for normal-hearing participants than for CI users (Mühler et al., 2008). Logatomes are often used for comparisons of recognition performance of automatic speech recognizers, emphasizing the sensitivity to small spectral and temporal differences between logatomes (Meyer et al., 2011). Faster-acting AGC systems, by narrowing the DR of a signal, can reduce the temporal variations within a logatome. Less salient differences can impair the intelligibility of more complex words and sentences (Rahne et al., 2010). A logatome test therefore represents a good measure to compare different sound processing schemes that affect features of the signal such as its DR.

Using a fast-acting AGC system with more than one band (i.e., a multichannel AGC) includes advantages and disadvantages (see Stone et al., 1999 for a summary). Advantages include a restoration of loudness perception (that requires extensive loudness growth function measurement), recruitment compensation, and restoration of the audibility of soft sounds that follow loud sounds. However, disadvantages include under- and overshoot effects of the temporal envelope, possible distortion to features such as formants sliding between bands, reduced intensity contrasts, and a flattened spectrum that may have a detrimental effect on speech intelligibility. In a CI simulation, Stone and Moore (2003, 2004, 2007) found that fast-AGC processing degraded intelligibility in the presence of a competing talker. This was partly due to cross modulations introduced by the AGC, fluctuations in the level of one talker correlated with fluctuations in the level of the other talker, and therefore a decreased ability to segregate the two talkers. Instead of a fast-acting multiband AGC system, a slow-acting alternative could avoid the drawbacks described earlier. The CI manufacturers Advanced Bionics, Cochlear, and MED-EL all use a slow-acting AGC system.

Multichannel compression can provide speech perception benefits as described earlier. However, these benefits may be constrained by the large compression ratio (the amount of reduction per magnitude step) used in CIs to convert the acoustic envelope into electric pulses, the acoustic-to-electric (ATE) channel mapping function. For this reason, this study investigates a dynamic compensation system based on the xDP output compression strategy by Oticon Medical (Bozorg-Grayeli et al., 2016), which uses a slow-acting multiband, compression design with adaptive compression curves for the ATE channel mapping and compares the performance of this system with that of a front-end single-channel dual-loop AGC system with linear ATE mapping. Speech intelligibility and logatome perception act as measures of perceptual performance for this comparison, while a measure of predicted intelligibility and electrode analysis is used for an objective analysis of both systems.

## Methods

### Participants

Ten postlingually deafened adult users of the Neuro Zti/Neuro One CI system (Oticon Medical, Vallauris, France) were included in this study (see Table 1). All measures were performed at the ENT department of the Medical University Hannover during routine patient visits. All participants, who had a minimum of 4 months of experience with their CI and at least 60% speech intelligibility in quiet, as well as 20% in noise at +10 dB speech-to-noise ratio (SNR) using the Hochmair–Schulz–Moser (HSM) sentence test, routinely underwent additional measurements described later. During the experiment, participants were offered regular breaks and were free to request a break at any time. The participants were measured via the Auxiliary In of the Neuro One sound processor connected to a standard PC. The order of the tests, settings, and conditions described in the following paragraphs were pseudorandomized across patients to avoid any training effect.

**Table 1.** Demographic details of the 10 Participating CI listeners.

ID	Age (years)	Gender	Duration of deafness (years)	Aetiology	CI usage (months)
CI01	36	f	2	Acute	27
CI02	75	f	1.5	Acute	5
CI03	62	f	1.5	Acute	8
CI04	71	m	1	Unknown	5
CI05	77	m	50	Eardrum perf.	4
CI06	36	m	3	Acute	9
CI07	35	m	35	Unknown	5
CI08	72	m	3	Unknown	6
CI09	59	m	55	Meningococcal	11
CI10	52	m	25	Traumatic	14

AVG	57.5		17.7		9.4
*SD*	16.0		20.7		6.6

*Note*. Average (AVG) and standard deviation (SD) presented for continuous variables. CI = cochlear implant.

### Sound Processing Settings

**Figure 1.**
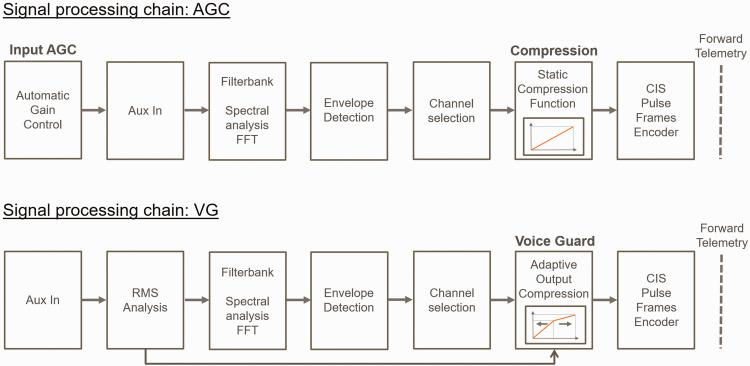
Signal Processing Chain for Both Processor Settings. AGC = automatic gain control; FFT = fast Fourier transform; RMS = root-mean-square.

A research CI sound processor was programmed with two different settings (see Figure 1). The first setting (AGC) was a single-channel dual-loop front-end AGC system implemented after Stone et al. (1999, DUAL-LO) with a knee-point at 63 dB SPL and a compression ratio of three above the knee-point for the slow component. In a dual-loop AGC system, a slow and a fast component are incorporated. The slow component is composed of a slow-averaging detector and compressor, controlling changes exceeding durations of hundreds of milliseconds. For this component, the attack and release times were 325 ms and 1,000 ms, respectively. For the fast component, the peak detector that focuses on unexpected loud sounds such as transients, a fixed knee-point of 71 dB SPL with a compression ratio of 12 and a 5 ms attack time was used with a 75 ms release time. The AGC compressor was implemented and executed in MATLAB (Version 2012b, The Mathworks, Natick, MA, USA) run on a computer. After processing, the signal was delivered to the Auxiliary In and processed further according to the Crystalis sound coding strategy. A standard spectral analysis via an FFT-based filter bank and an envelope detector was used for the analysis to choose eight filter channels that had the highest envelope magnitude across the filter channels, which would stimulate with a given phase duration (“NofM” strategy; Seligman & McDermott, 1995). A static linear compression function was used to map sound amplitude in dB to pulse width in µs. This linear ATE mapping function was not clinical standard and was used for deactivating the usually compressive mapping function used in CIs by Oticon Medical. The Crystalis sound coding strategy is very different from the conventional amplitude modulation of biphasic pulses. It modulates pulse width (duration) to reflect changes in the amplitude according to the envelope energy of each associated bandpass filter (the pulse height is fixed and the width changed according to the magnitude of the envelope associated with the stimulating electrode). The stimulation rate can be adjusted from 150 to 1,000 pulses per second. The default clinical setting of 500 pulses per second was used by every participant. Finally, the information was processed in the Continuous Interleaved Sampling (CIS)-like pulse frame encoder (Wilson et al., 1991) to deliver the pulses to the electrode array.

**Figure 2.**
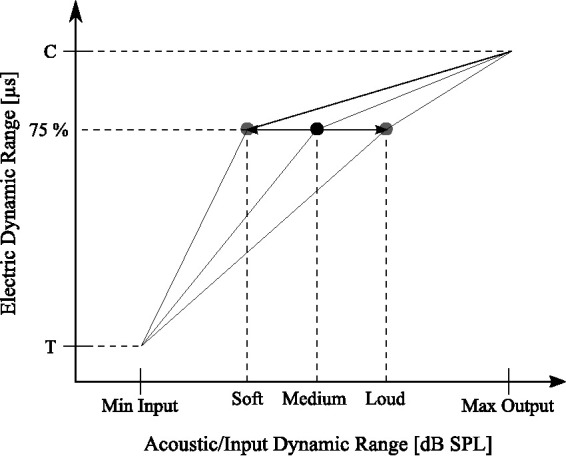
Principle of the Back-End Dynamic Compression System Voice Guard and its ATE Mapping With Electric Dynamic Range as a Function of Acoustic Dynamic Range. The three presets *Quiet*, *Medium*, and *Loud* correspond to the RMS input level and knee-point values found in Table 2. SPL = sound pressure level.

The second setting was Voice Guard (VG, depicted in the lower panel of Figure 1). In this setting, the root-mean-square (RMS) of the broadband input signal was estimated for the VG settings. For the RMS calculation, the signal was sampled at 500 Hz with the time domain analysis being averaged over a rectangular 2 s window in each band. After the RMS analysis, the FFT filter bank, envelope detection, and channel selection were performed as in the first setting. Unlike the first setting, adaptive ATE compression functions (effectively gains for the individual electrodes) derived from the RMS analysis were used to map the acoustic levels in dB to the pulse duration in µs for the specific electrodes (see Adaptive Output Compression in Figure 1 and the ATE mapping principle in Figure 2). Table 2 describes the algorithm. The knee-point adapted on the mean RMS and was updated every 2 ms. There were 14 equally spaced steps between the *Quiet* and *Loud* presets. Higher knee-point values were set for the low-frequency bands (emphasizing the low frequencies for the CI listener, as ATE compression was applied at lower input levels in the high-frequency bands). The amount of compression applied depended on the electric DR of the CI user. The input/output compression characteristic was always compressive; however, the amount of compression increased above the knee-point. The knee-points were computed such that 95% of the sound intensity was mapped below the knee-point for a particular RMS input level for clean speech (see Table 2). Knee-points were mapped to 75% of the electric DR, based on the assumption that speech understanding is optimal for CI users at these stimulation levels. The Crystalis sound coding strategy with the same stimulation rates as in the AGC setting was used.

**Table 2.** Voice Guard Settings: Three examples of RMS input levels and their corresponding Knee-Point Values for each frequency band.

Environment	Quiet	Medium	Loud	Frequency range [Hz]	Allocated electrodes
RMS input level (dB SPL)	60	70	80	–	–
Knee-point value (dB SPL)	52	61	70	195–846	16–20
	52	61	70	846–1497	11–16
	47	57	66	1497–3451	6–11
	41	50	58	3451–8000	1–6

*Note*. Table adapted from Segovia-Martinez et al. (2016). RMS = root-mean-square; SPL = sound pressure level.

Both ATE settings were set to an IDR of 35 to 85 dB SPL, which deviated from the standard range of 25 to 95 dB SPL the participants used in their clinical setting. The experimental IDR resembled the DR used by most commercially available CI devices. Signal levels below this range were ignored, and those above were compressed with an infinite compression ratio acting as a limiter. The threshold and most comfortable levels of the participant’s clinical setting were used for both settings. Both settings were loudness balanced by using the AGC setting as reference for a 65 dB SPL input level (which always resulted in a comfortable loudness) and switching between settings after the presentation of five randomly selected HSM sentences and asking the participant if the reference had the same loudness as the other setting. The HSM sentences used for this purpose were not used in the actual testing. All participants used VG as their everyday clinical setting, the only differences being the reduced IDR and the presentation over the Auxiliary Input instead of the sound processor microphones.

### Behavioral Performance

Speech intelligibility was assessed with the HSM sentence test, consisting of everyday sentences with various length and complexity (Hochmair-Desoyer et al., 1997). Two conditions for each setting were measured: in quiet and in noise. A cafeteria noise from the D-CASE 2013 database (Stowell et al., 2015) was used at an SNR of +10 dB. The noise preceded the sentences for 3 s and ended 1 s after presentation. One training list was presented followed by two test lists that were averaged for the speech intelligibility result for each of the settings.

Logatome perception was determined with a consonant recognition task performed with the MACarena speech test (Umat et al., 2006). Twelve consonant logatomes (“aPa,” “aTa,” “aKa,” “aBa,” “aDa,” “aGa,” “aMa,” “aNa,” “aLa,” “aRa,” “aFa,” and “aSa”) containing different phonetic cues were randomly presented four times for a total of 48 trials. Participants indicated their response via a graphical user interface displaying all 12 consonants as labeled buttons. In the beginning, a familiarization phase was performed in which the participant could click on each button and listen to the logatome for as long as desired. Normally, the examiner stopped this phase either after 5 minutes or after receiving confirmation from the participant. Next, a training phase included the presentation of all 48 trials during which participants could repeat each logatome. Visual feedback was provided after each response of the participant. Finally, the test phase consisted of the presentation of all 48 trials to which participants entered their answers on the screen. Logatome perception was only assessed in quiet. The whole procedure was performed for both strategies, and the starting strategy was randomized across participants. Phonetic cues (Frication, Nasality, Voicing) describe the characteristics of spoken sounds and can be associated with different consonants used in the logatome test, as described by Dillier et al. (1993).

The information transmission from input to output in bits per stimulus is a measure of covariance between input and output described by Miller and Nicely (1955). It was used in this study as a performance measure specific to the different phonetic cues. The mean logarithmic probability (MLP) for input, output, and the joint occurrence of both is used for the information transmission computation and defined as

(1) $$\mathrm{MLP}\left(x\right)=E\left(-\log\;p_{i}\right)=-\sum_{i}{\;p}_{i}\;\log\;p_{i}$$

with *x* as input variable assuming discrete values *i* = 1, 2, … , *k* and $p_{i}$ its probability, estimated from the correct responses relative to the number of total presentations obtained in the experiment. The number of bits of information per stimulus is achieved when the logarithm of Equation 1 is taken to the base 2. The same equation holds for the output variable *y*, which can hold the values *j* = 1, 2, … , *m*. The measure of covariance of input with output (also called transmission from *x* to *y,* in bits per stimulus) is given by

(2) $$T\left(x;y\right)=\;\mathrm{MLP}\left(x\right)+\mathrm{MLP}\left(y\right)-\mathrm{MLP}\left(xy\right)=-\sum_{i,j}\;p_{ij}\log\frac{p_{i}p_{j}}{p_{ij}}$$

with *MLP(xy)* as the number of decisions needed to specify the particular stimulus-response pair and $p_{ij}$ as the joint occurrence probability of input *i* and output *j*. The relative transmission *T_rel_* used as output measure in this study and ranging from 0 to 1 is achieved by

(3) $$T_{\mathrm{rel}}\left(x;y\right)=T\left(x;y\right)/H\left(x\right)$$

with $H\left(x\right)$ as the expected value of information from source *x* and the relationship $H\left(x\right)\geq T\left(x;y\right)\geq 0$. For more details, see Miller and Nicely (1955).

## Results

### Speech Intelligibility

**Figure 3.**
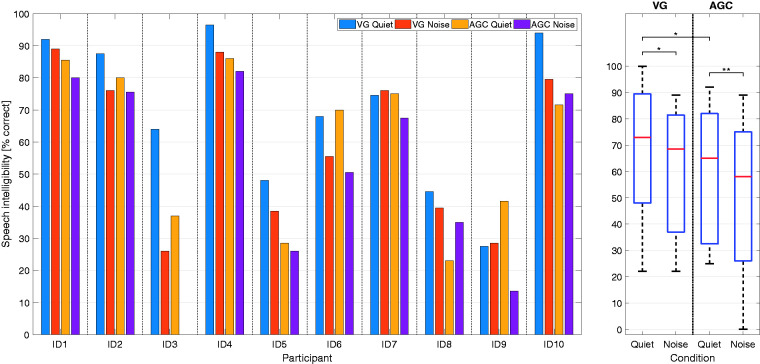
Individual Speech Intelligibility Results for All Participants, Conditions, and Settings (Left) as well as Their Summarizing Box Plot Representations (Right). The median is in red, edges show the 25th and 75th percentiles, and whiskers the most extreme data points. Significance symbols indicate *p* < .05 with * and *p* < .01 with **. VG = Voice Guard; AGC = automatic gain control.

Figure 3 shows the individual speech intelligibility results of all participants on the left panel, while the right panel shows the results in the form of a box plot for both settings and conditions. A repeated-measures analysis of variance found that the setting (AGC vs. VG) — *F*(1, 9) = 7.373, *p* = .031 — and the conditions (quiet vs. noise) — *F*(1, 9) = 14.250, *p* = .008 — had a significant effect on the speech intelligibility outcome. No significant interaction between the setting and condition was found, *F*(1, 9) = 0.034, *p* = .858. After it was found that the data were normally distributed, a paired-sample *t* test with Bonferroni correction applied was conducted to compare speech intelligibility between conditions and settings. For speech-in-quiet, a significant difference between the VG (mean [*M*] = 69.6%, standard deviation [*SD*] = 23.7%) and AGC (*M* = 59.9%, *SD* = 24.7%) settings was found, *t*(9) = 2.827, *p* = .041. There was no significant difference between the settings for speech in noise, *t*(9) = 1.966, *p* = .081. Speech perception performance was significantly better in quiet than in noise both with the VG and AGC settings — *t*(9) = 2.426, *p* = .024 for VG and *t*(9) = 3.904, *p* = .031 for AGC.

### Logatome Perception

Table 3 shows the information transmission results averaged over the seven participants who could perform this test (namely, ID01, 03, 04, 06, 08, 09, and 10 from Table 1). The three remaining participants could not perform this test due to time constraints in the clinical routine. A repeated-measures analysis of variance with factors setting and phonetic cue revealed that the setting had no significant effect on the logatome perception (AGC vs. VG), *F*(1, 6) = 4.400, *p* = .081. However, the phonetic cue had a significant effect on speech intelligibility, *F*(6, 36) = 4.056, *p* = .005. No significant interaction between the setting and phonetic cue was found, *F*(6, 36) = 1.703, *p* = .148.

**Table 3.** Relative transmission results from equation 3 of seven participants who completed the logatome test.

Relative transmission	AGC	VG
Overall	0.61	0.70
Voiced	0.48	0.59
Nasality	0.37	0.38
Sonorance	0.47	0.59
Sibilance	0.42	0.69
Frication	0.16	0.35
Place	0.17	0.19
Manner	0.48	0.57

*Note*. Except the overall results, all other results correspond to specific phonetic cues. AGC = automatic gain control; VG = Voice Guard.

In addition, averaged confusion matrices generated from the results of all participants were evaluated with the performance measures accuracy (overall effectiveness) and the accuracy score (F1 score). Accuracy is obtained by the sum of true positives and true negatives divided by the number of answers of the participants. The F1 score is obtained by precision (the number of true positives divided by the number of all positives returned by the participant) and recall (the number of true positives divided by the number of all samples presented). The accuracy was 0.43 and 0.52 and the F1 score was 0.44 and 0.51 for the AGC and VG setting, respectively.

### Short-Time Objective Intelligibility Measure

**Figure 4.**
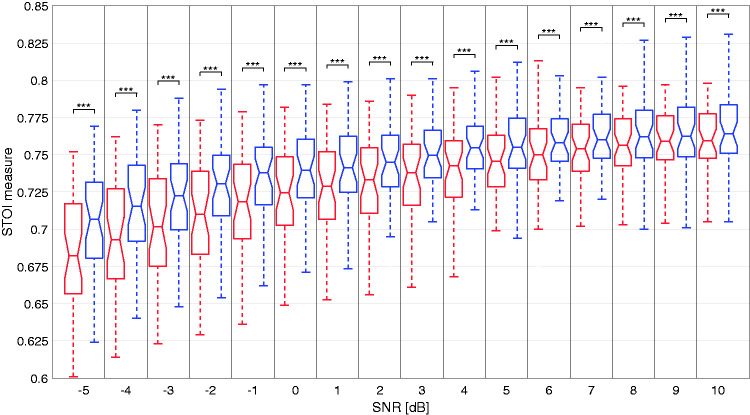
Boxplot of the STOI Measure as a Function of SNR for the AGC (Red) and VG (Blue) Setting. The median is the line between 25th and 75th percentiles marked as the edges of the box plots. Outliers were excluded for simplicity. *** denote a significant difference with *p* < .001. STOI = Short-Time Objective Intelligibility; SNR = speech-to-noise ratio.

The Short-Term Objective Intelligibility (STOI) measure performs a spectrotemporal cross-correlation of a clean speech signal with its degraded/noisy counterpart (Taal et al., 2012). This measure, ranging in its output from zero to one, shows the resemblance of the degraded signal to its reference and therefore a measure of intelligibility. To perform this analysis, 40 sentences of the HSM sentence test (two lists) were selected at SNRs ranging from –5 to 10 dB with the same cafeteria noise as used in the speech intelligibility task. To compare the VG and AGC setting, a software framework from Oticon Medical — a collection of MATLAB functions to process wav files according to the commercial device with the freedom to alter almost every detail — was used to process each file with its corresponding setting and subsequently use a sine-carrier vocoder to generate the final degraded speech material. The sine-carrier vocoder converted the resulting pulse width range to a dB SPL range, and each pulse was used to modulate the amplitude of standard sine waves, with the frequency of the sine waves corresponding to the center frequency of each FFT filter. The threshold and most comfortable levels were fixed for both settings and derived from the average of all participants. Figure 4 shows the STOI measure for the AGC (red) and the VG (blue) setting as a function of SNR. A paired-sample *t* test with Bonferroni correction for multiple comparisons identified a significant difference for all SNRs between the AGC (*M* = 0.733, *SD* = 0.038 across all SNRs) and VG (*M* = 0.746, *SD* = 0.033 across all SNRs) setting with *p* < .001. The difference between settings was larger at negative SNRs compared with positive SNRs.

### Electrodogram Analysis

Two additional descriptive objective measures of the electrodograms, generated with the experimental framework by Oticon Medical, were analyzed for all SNRs: (1) the SNR output (${\mathrm{SNR}}_{\mathrm{OUT}})$ similar to Nogueira et al. (2016) and Khing et al. (2013a) from the averaged RMS power of the electrodogram obtained for the speech signal alone divided by the RMS power for the noise signal (cafeteria noise used for the speech intelligibility task) alone:

(4) $${\mathrm{SNR}}_{\mathrm{OUT}}=\;10\cdot \log_{10}\left[\frac{\sum_{t=1}^{n}\sum_{C=1}^{20}{E_{S}\left(t,C\right)}^{2}}{\sum_{t=1}^{n}\sum_{C=1}^{20}{E_{N}\left(t,C\right)}^{2}}\right]$$

with $E_{S}\;$and $E_{N}$ being the electrodograms for the speech and the noise, *t* the time, and *C* the electrode in the electrodogram. The equation assumes linearity in the operations conducted by the sound coding strategy, which in reality is not the case because of different compression stages and operations such as NofM. The work around suggested by Nogueira et al. (2016) multiplied the results from the FFT filter bank with the gains from the ATE mapping of the VG algorithm, which is a linear operation but keeps the effect of VG. In the case of AGC, a linear ATE mapping function was used, so only the FFT results were extracted to generate the SNR outputs. The whole electrodogram was used for the RMS power analysis. Twenty HSM sentences were used for the analysis. The range of input SNRs (SNR_IN_) was generated by keeping the speech at a digital level corresponding to 65 dB SPL and the noise at input levels ranging from 55 to 70 dB SPL (the wave files were resampled and rescaled to dB FS by an internal scaling factor). (2) A percentile analysis was performed by comparing the 33^rd^, 66^th^, and 99^th^ amplitude percentiles of the electrodogram of each setting with the International Speech Test Signal (ISTS; Holube et al., 2010) at 65 dB SPL input level, relating to the international standard IEC 60118-15:2012 for measuring speech for air-conduction hearing aids. For an easier analysis, pulse widths were converted into equivalent charge and the percentile analysis performed across the electrodogram. The percentile analysis was performed to achieve an understanding of each setting’s resulting DR.

**Figure 5.**
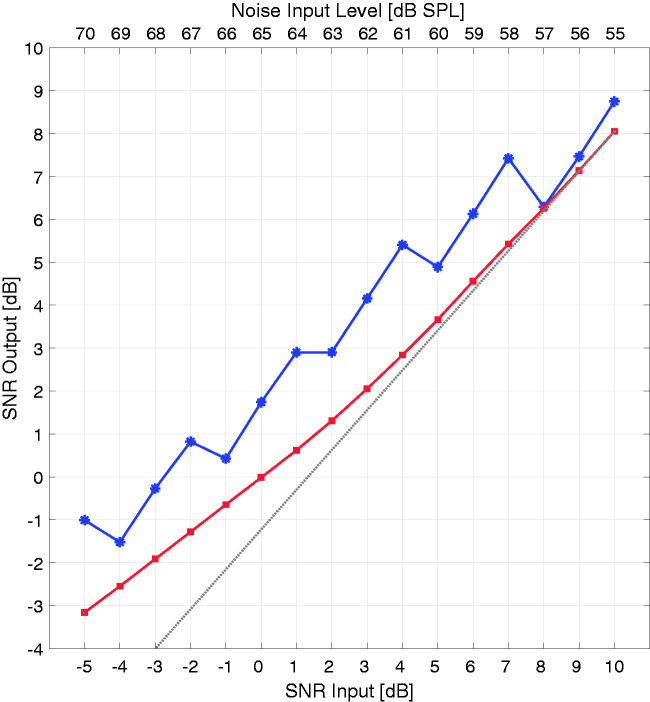
SNR_OUT_ According to Equation 4 as a Function of Input SNR/Noise Input Level in dB SPL for the AGC (Red) and VG (Blue) Setting. The dotted gray line helps to highlight the AGC compression setting with a knee-point at 63 dB and a compression ratio of 3 for the slow component of the DUAL-LO AGC. SNR = speech-to-noise ratio; SPL = sound pressure level.

Results presented in Figure 5 show that the SNR_OUT_ is higher across all input SNRs for the VG setting compared with AGC. While the AGC setting shows a consistent increase with approximately 0.92 dB/SNR_IN_, the VG setting results in a step-like increase of 0.68 dB/SNR_IN_ when both are fitted linearly. We hypothesize that the signal at specific input levels exceeds the compression threshold across the four bands in the VG algorithm, which attenuates the signal similarly and results in the coarse progression (compare, e.g., noise input level at 66 and 67 dB). Overall, when processing the speech and noise signals individually, the analysis shows that VG creates a higher SNR_OUT_ than AGC across all input levels.

**Figure 6.**
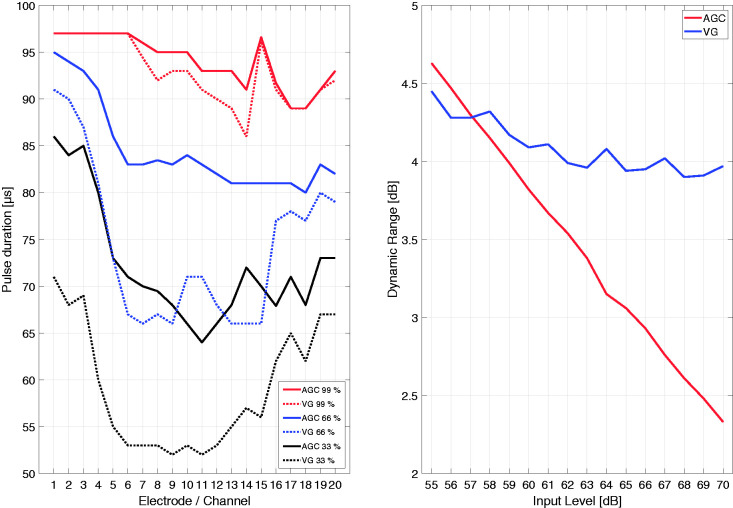
A: Percentile analysis for the 33^rd^ (black), 66^th^ (blue), and 99^th^ (red) percentile for both settings, displayed as pulse duration as a function of electrode number. B: Averaged dynamic range in dB across input levels in dB for the ISTS as input signal. ISTS = International Speech Test Signal; AGC= automatic gain control; VG = Voice Guard.

Figure 6 displays the percentile analysis, that is, the pulse duration plotted as a function of the electrode number. The dotted lines show the 33^rd^ (black), 66^th^ (blue), and 99^th^ (red) percentiles of the VG setting, while their solid-line counterparts show the same percentiles but for the AGC setting. The ISTS had a duration of 9 s. For example, the 33^rd^ percentiles are the pulse duration below which 33% of the pulse durations may be found. The left panel of Figure 5 displays the percentiles, whilst the right panel plots the DR in dB derived from the difference between the 99^th^ and 33^rd^ percentiles. The left plot shows that the pulse duration for the 33^rd^ and 66^th^ percentiles is lower for the VG setting than the AGC setting. This is the case for almost all electrodes except for the high frequencies (i.e., electrodes 16–20). Below these electrodes, the average difference in pulse duration between settings is around 15 µs. The 99^th^ percentiles, which can be regarded as the most comfortable level of each individual electrode during stimulation, are similar between settings across all electrodes. Only small differences between VG and AGC of around 5 µs can be seen between electrodes 6 and 14. The right panel of Figure 5 shows that, for low input levels, the DR of each setting is similar. However, when increasing the input level, the DR of the AGC setting decreases constantly, while the VG setting stays at around 4 dB for all input levels.

## Discussion

This study compared a single-channel dual-loop front-end AGC with linear ATE mapping against an adaptive back-end compression system by measuring speech and logatome intelligibility, alongside objective measures of intelligibility. For speech intelligibility in quiet, a significant benefit with VG over AGC of 10 percent points was observed. For the noise condition, no significant difference was found. Logatome perception, although not statistically significant, increased by 9 percent points when using VG compared with AGC (this improvement did not reach statistical significance probably due to the small number of seven participants). The objective analysis of speech in noise based on STOI and the electrodogram analysis (SNR Input/Output and percentiles) indicated a benefit for the VG setting compared with single-channel front-end compression.

The aforementioned ADRO algorithm has been shown to improve speech intelligibility in quiet and in noise in several studies (Blamey, 2005; Blamey et al., 2005; Dawson et al., 2004). The main differences between ADRO and the VG processing is the number of effective bands and the order in the signal processing chain (see Table 4). While ADRO is using compression on each individual filter (22 for the Cochlear CI system), VG is using four frequency bands, each comprised of up to six electrodes (see Table 2). ADRO is used before the ATE mapping which includes a compressive function and VG effectively is the ATE mapping in the processing. In the five studies reported in Blamey (2005), three studies tested hearing aid users with the algorithm, while the other two were performed at low intensities, an area in which the most improvements with such an algorithm are expected (Firszt et al., 2004). Therefore, an actual comparison cannot be made between both outcomes. In our study, we found a significant improvement of 10% of speech intelligibility in quiet, but no improvement of speech intelligibility in noise. However, an acute testing reduces the full effectiveness of the new setting due to a lack of familiarization (Dorman & Loizou, 1997; Fu, 2002; Vermeire et al., 2010). As all participants used the VG setting as their standard clinical setting and were not familiar with the AGC setting, more experience with the AGC setting could have led to greater speech intelligibility. However, the objective measurements suggest a benefit of VG over AGC. In addition, the likelihood of a change in performance over time when changing between front-end and back-end compression is rather small. We suspect that the change in, for example, a stimulation mode, would rather show an effect over time.

**Table 4.** Comparison of the signal processing principles ADRO, envelope profile limiter and VG.

	Signal processing principle		
Aspect	ADRO	Envelope profile limiter	VG
Location in signal processing path	After envelope detector, before ATE	After envelope detector, before ATE	ATE

Number of bands	22	22	4

Time constants	Rule based	Attack = 0 ms Release = 625 ms	Attack = 2 s Release = 18 ms

Knee-points	Percentile analysis-based rules	Percentile analysis-based rules	Depending on RMS

*Note*. ADRO = Adaptive Dynamic Range Optimization; VG = Voice Guard; ATE = acoustic-to-electric; RMS = root-mean-square.

The statistical analysis of the relative transmission resulting from the logatome perception task described in Equation 3 revealed a significantly better performance with the VG setting. From the tested phonetic cues, only one significant difference for the fricatives represented by the consonants “s” and “f” used in the measurement could be seen. Both consonants inhibit the *fortis* (latin for strong) attribute in linguistics, meaning a stronger spoken consonant and a higher energy concentration than the ones described with *lenis* (latin for soft, e.g., “b” and “w”; Ladefoged & Maddieson, 1996). This implies that logatomes with a greater energy as well as high frequency content can be identified well when the VG DR of the signal is larger compared with a standard front-end AGC. However, the multichannel aspect may explain the potential improvement of VG. A DR preservation by VG in the logatome task can be ruled out, because the knee-point changes with a duration of up to 2 s are too slow to influence the perception of logatomes. The AGC processing used a preemphasis before the actual AGC, which attenuated the amplitude of low frequencies, but not of high frequencies. This means that the AGC preserved the fricatives, and therefore, participants were likely to perceive the relevant consonant.

The STOI measure showed a significantly stronger correlation between the degraded and the clean speech signal with the VG setting than with the AGC setting across all SNRs, although there was an average difference of only 0.013 points. This difference may result from the sine-carrier vocoder reducing the available information drastically for both settings. The non-significance that is present in the noise condition between AGC and VG can be linked to the small difference in the STOI results between both settings at +10 dB SNR. Although the latter is statistically significant, the medians and percentiles are closer together when compared with the –5 dB SNR condition. This implies that a general improvement of VG over AGC was not apparent with the STOI measure, consistent with the speech intelligibility results in noise.

The objective SNR analysis suggests that the signal processing, assuming the linearity required for the analysis technique, results in better performance with the VG setting. This is shown with a positive SNR_OUT_ reached at more difficult SNRs for the VG processing (at –2 dB SNR_IN_) than for the AGC processing (at +1 dB SNR_IN_). This is consistent with the results from the STOI measure and the small but not significant speech-in-noise outcome, showing smaller differences in SNR_OUT_ for higher input SNRs. While the AGC setting showed a smooth transition across input SNRs, the VG setting results in a step-like course, with some input SNRs resulting in the same SNR_OUT_. In addition, the SNR is squeezed more in the VG processing than in the AGC processing as reflected in the slope of both curves in Figure 5. As mentioned earlier, the adaptive processing of the VG algorithm, when increasing the input level, may center on the effective DR of the signal (just like the compression principle of the ADRO algorithm). This would explain the unusual input/output function of VG compared with that of the AGC setting. The small difference in SNR_OUT_ between AGC and VG at +10 dB SNR is also reflected in the behavioral measurements at that particular SNR (see Figure 3).

The percentiles shown in Figure 5 can be interpreted as a measure of the DR used for stimulation with the VG or AGC setting. Comparing AGC and VG show that the latter setting features a broader DR when processing the ISTS signal, especially at higher ISTS input levels. The constant DR resulting from the ATE mapping of the VG shows the processing of the adaptive compression functions. Comparing the difference in pulse duration for the example shown in the left plot of Figure 5 between the 99^th^ and 33^rd^ percentile across electrodes the AGC setting results in 27.2 µs and VG in 32.6 µs on average. The pulse width DR of the participants as set in their respective fitting is around 140 µs. The average pulse width derived from our analysis suggests that VG uses more of the DR of the fluctuating ISTS speech signal compared with AGC, but the difference between both is rather small when compared with the DR of the clinical fitting.

In summary, this study revealed some benefits of an adaptive back-end dynamic compensation system compared with a standard front-end AGC system with linear ATE mapping. VG performed better for speech-in-quiet, at logatome perception (overall, essentially because of the fricatives “aSa” and “aFa”) and showed a higher STOI measure. The objective measures show that the adaptive knee-points of the VG algorithm better preserve the dynamics of speech signals but alter the absolute dynamics of the sound signals. This study compared a single-channel system with a multichannel system. It would be interesting to compare the VG processing with a multichannel front-end AGC system and explore the effectiveness of the analysis part of the signal processing chain.
